# Omega-3 docosahexaenoic acid induces pyroptosis cell death in triple-negative breast cancer cells

**DOI:** 10.1038/s41598-018-20422-0

**Published:** 2018-01-31

**Authors:** Nathalia Pizato, Beatriz Christina Luzete, Larissa Fernanda Melo Vasconcelos Kiffer, Luís Henrique Corrêa, Igor de Oliveira Santos, José Antônio Fagundes Assumpção, Marina Kiyomi Ito, Kelly Grace Magalhães

**Affiliations:** 1 Department of Nutrition, University of Brasilia, Brasilia, 70910–900 Brazil; 2 Laboratory of Immunology and Inflammation, University of Brasilia, Brasilia, 70910-900 Brazil

## Abstract

The implication of inflammation in pathophysiology of several type of cancers has been under intense investigation. Omega-3 fatty acids can modulate inflammation and present anticancer effects, promoting cancer cell death. Pyroptosis is an inflammation related cell death and so far, the function of docosahexaenoic acid (DHA) in pyroptosis cell death has not been described. This study investigated the role of DHA in triggering pyroptosis activation in breast cancer cells. MDA-MB-231 breast cancer cells were supplemented with DHA and inflammation cell death was analyzed. DHA-treated breast cancer cells triggered increased caspase-1and gasdermin D activation, enhanced IL-1β secretion, translocated HMGB1 towards the cytoplasm, and membrane pore formation when compared to untreated cells, suggesting DHA induces pyroptosis programmed cell death in breast cancer cells. Moreover, caspase-1 inhibitor (YVAD) could protect breast cancer cells from DHA-induced pyroptotic cell death. In addition, membrane pore formation showed to be a lysosomal damage and ROS formation-depended event in breast cancer cells. DHA triggered pyroptosis cell death in MDA-MB-231by activating several pyroptosis markers in these cells. This is the first study that shows the effect of DHA triggering pyroptosis programmed cell death in breast cancer cells and it could improve the understanding of the omega-3 supplementation during breast cancer treatment.

## Introduction

Triple-negative breast cancer (TNBC) represents from 10 to 20% of all breast carcinomas. It refers to breast cancers that do not express genes for estrogen receptor, progesterone receptor and epidermal growth factor receptor 2 and do not respond to therapies targeted to these receptors^1,2^. This type of breast cancer is more aggressive and has higher recurrence and death rates than other subtypes in the first years after treatment^3,4^. MDA-MB-231 human cells express this triple-negative immunoprofile and are the main cell line used to investigate this breast cancer subtype^5,6^. They are flattened and spindle-like cells with increased cell-cell contacts and an aggressive phenotype^7^. Moreover, the 4T1 murine cells are also typical triple negative breast cancer cell line and closely mimic human breast cancer considering characteristics as anatomical site, immunogenicity and growth development.

Docosahexaenoic acid (DHA) is an omega-3 fatty acid that has anticancer effects. Studies *in vitro* documented that DHA can inhibit breast cancer cell growth and increase apoptosis^8–11^ as well as reduce cell invasiveness potential^12^. It has been shown that DHA attenuated breast cancer progression and lung metastasis by suppressing metalloproteinases. On the other hand, arachidonic acid (AA) is a fatty acid of the omega -6 family and is associated with growth and tumor progression^13–17^.

Different forms of cell death are known such as apoptosis^18^, necroptosis^19^, pyroptosis^20^ and pyronecrosis^21^. Among them, apoptosis is the most completely described pathway until now and the only one related to the breast cancer cell death induced by DHA^8–11^. However, no study has yet assessed the DHA-induced action on cell death towards pyroptosis pathway.

Pyroptosis was first observed in macrophages infected with *Shigella flexneri*^22^ but it can occur in several other cell types^23,24^ and be activated by a variety of stimuli other than the presence of infection^23,25^. This form of cell death is a programmed process mediated by proteases so-called inflammatory caspases (caspases-1, -4, -5 in humans and -11 in mice) activated within inflammasomes, that will culminate to pore formation in cell membrane as the most important event in pyroptosis. The key molecule that lead to pore formation in cell membrane is Gasdermin D (53 kDa) that is cleaved by these caspases into a N-terminal (31 kDa) and C-terminal (22 kDa) fragments. The N-terminal fragment will target, insert and permeabilize cell membranes forming pores^26–28^.

Caspase-1 is activated by inflammasome or pyroptosome and leads to processing and subsequent secretion of interleukin-1β (IL-1β) and IL-18 inflammatory cytokines as well as high mobility group box 1 protein (HMGB1)^29–31^. Furthermore, it induces pore formation, cell membrane rupture and intracellular proinflammatory contents release^32^. Among all inflammasomes, NLRP3 is the best described inflammasome until now and the mechanisms of its activation are not completely understood^33,34^. NF-κB is required on a priming step of activation and different stimulus can act as a second step to allow this multicomplex protein assembly^35^. The NLRP3 activators are proposed to act via different pathways like lysosomal damage^34^, reactive oxygen species (ROS) production^33^ and cytosolic K^+^ efflux^36^. Nuclear receptors can also regulate inflammasome activation in different ways. It has been show that nuclear receptors the orphan nuclear receptor small heterodimer partner (SHP) is a negative regulator of NLRP3 inflammasome activation^37^. Contrarily, agonism of the pregnane X receptor (PXR), another nuclear receptor, triggered the activation of NLRP3 inflammasome and the ensuing cleavage and maturation of caspase-1 and interleukin-1β (IL-1β)^38^. In addition, the agonism of another nuclear receptor, liver X receptors (LXRs) can trigger pyroptosis in colon cancer cells^39^.

The physiological role of pyroptosis is ambiguous. Infected cells can be eliminated by pyroptosis process as an important instrument for host defense against intracellular pathogens^40^. However, pyroptosis is also involved in the TCD4+ lymphocytes cell death during HIV infection leading to immunodeficiency^41^. Nevertheless, the relevance of pyroptosis in the context of omega-3 effect in breast cancer cells needs to be addressed. In this study, we hypothesized that pyroptosis activation could be a new anticancer mechanism of omega-3 DHA in breast cancer cells and investigated the signaling pathway involved in the induction of pyroptosis by DHA in triple-negative breast cancer cells MDA-MB-231.

## Results

### DHA decreased breast cancer cells but not non-cancer cells viability

**Figure 1:**
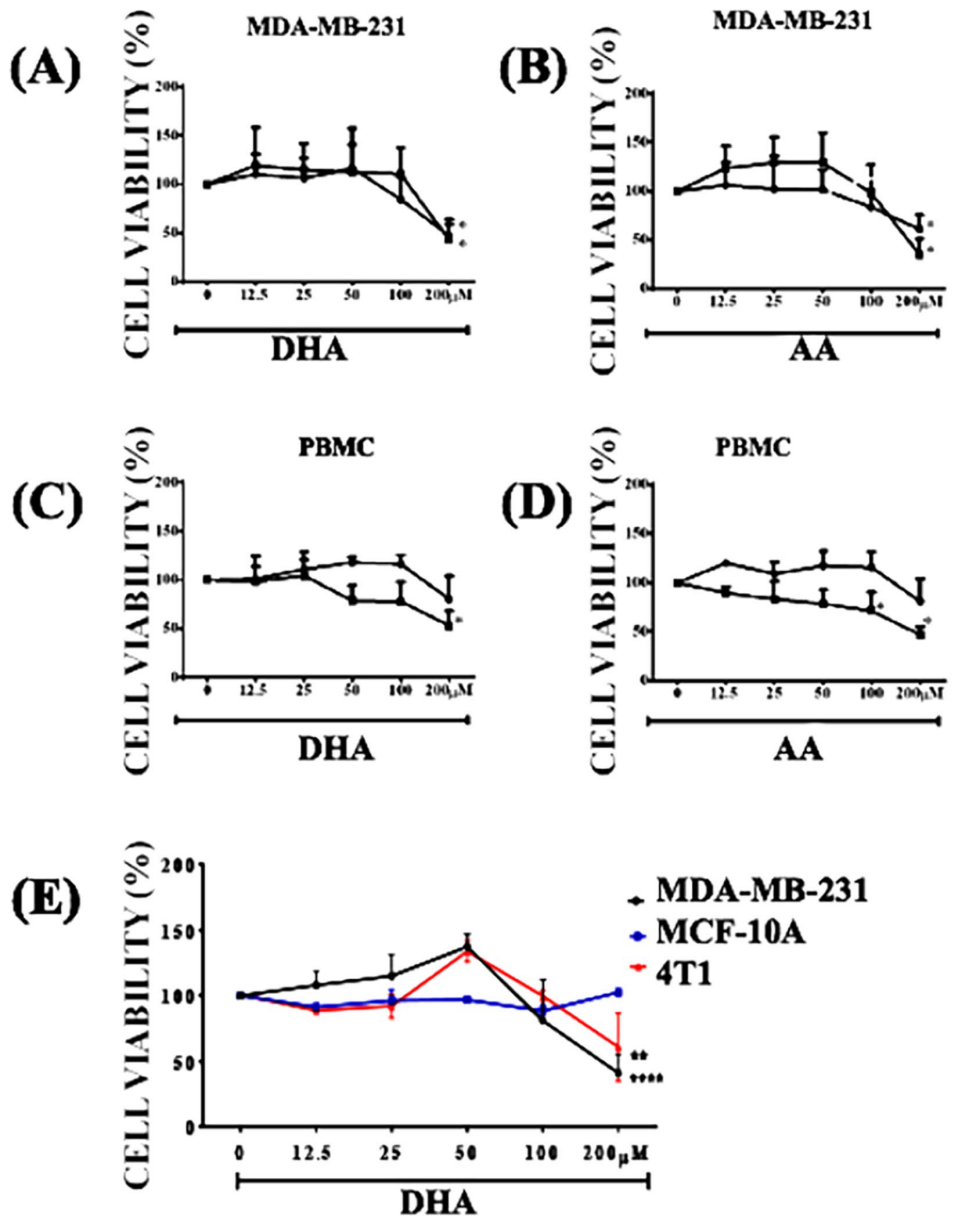
- DHA decreased cell viability of breast cancer cells (MDA-MB-231 and 4T1) but not normal mammary epithelial cells after 24 h of stimulation. MDA-MB-231 (**A**,**B**) and PBMC (**C**,**D**) were stimulated with different concentrations of AA or DHA for 24 h (lines with black circle) or 48 h (lines with black square) (**A**–**D**). Cell viability of the three different cell lines (MDA-MB-231, MCF-10A and 4T1) was also assessed after 24 h of stimulation (**E**) by MTT assay. The data are presented as percentage of cell viability relative to unstimulated cells (0 μM). Each point represents the average percentage ± SD. (n = 5) *p < 0.05 compared to unstimulated cells (0 μM).

To determine the DHA-induced cytotoxic effect in triple-negative breast cancer cells, we stimulated MDA-MB-231 cells with a range of different concentrations of DHA (12.5, 25, 50, 100 and 200 uM) and cell viability was assessed by MTT assay. DHA at 200 μM decreased 52.4% of MDA-MB-231 cell viability in 24 hours (Fig. 1A). This effect was not time or dose-dependent. Viable cells were decreased by 38,8% after treatment with AA at 200 μM for 24 h (Fig. 1B). The equivalent volume of the diluent was tested and caused no significant effect on cell viability (data not shown). DHA-induced cytotoxic effect was specific for breast cancer cells MDA-MB-231 and 4T1 (Fig. 1E) within 24 hours, since they did not alter cell viability in human peripheral blood mononuclear (Fig. 1C,D) or normal mammary epithelial cells MCF-10A (Fig. 1E) used here as controls. There was no difference between cytotoxicity effect of DHA (EC_50_ = 101.6 μM) and AA (EC_50_ = 109.3 μM) in MDA-MB-231 cells after 24 h of stimulation. However, the cytotoxic effect of DHA was much higher in breast cancer cells MDA-MB-231 (EC_50_ = 101.6 μM) and 4T1 (EC_50_ = 122.4 μM) when compared to non-cancerous cells MCF-10A (EC_50_ = 227.9 μM) and PBMC (EC_50_ = 188.6 μM) after 24 h of stimulation.

### DHA led to MDA-MB-231 cell death and induced pyroptosis markers

**Figure 2:**
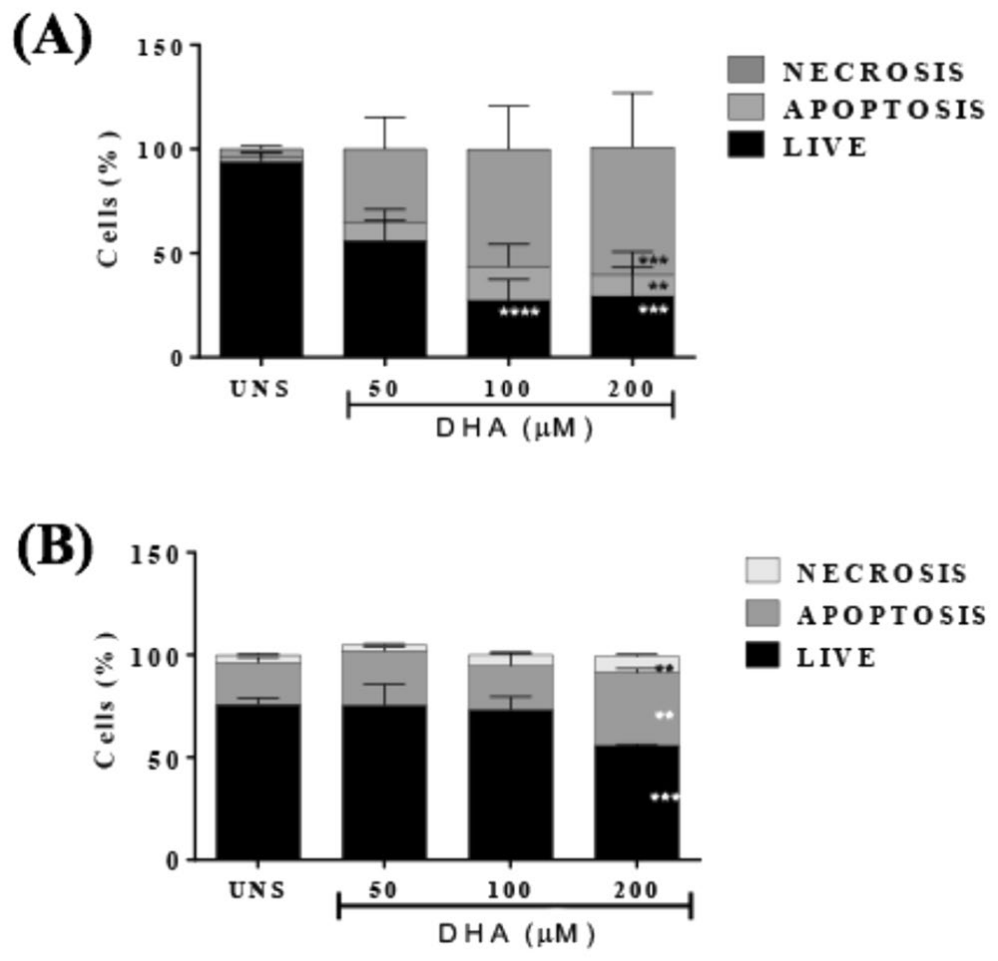
- DHA led to cell death in breast cancer cells. MDA-MB-231 (**A**) and 4T1 breast cells were stimulated with different concentrations of DHA for 24 h and cell death was assessed by labeling cells with annexin-V and propidium iodide followed by flow cytometry analysis. The percentage of necrotic, apoptotic or live cells is indicated. Data are representative of five independent experiments. UNS represents unstimulated cells. Each point represents the average percentage ± SD. (n = 5) *p < 0.05.

To investigate if the DHA-induced decrease in cell viability occurred by increased cell death, we executed annexin V/PI analysis. Treatment of MDA-MB-231 with 50, 100 and 200 μM of DHA for 24 h induced dose-dependent increase of both annexin+/PI+ cells in MDA-MB-231 cells (Fig. 2A) when compared to unstimulated cells. In contrast, DHA triggered an increase of annexin+/PI− cells in murine 4T1 cells (Fig. 2B) when compared to unstimulated cells. Since annexin+/PI+ cells represent necrotic condition characterized by membrane rupture, we decided to investigate whether PI + cells could be undergoing pyroptosis cell death and analyzed some cellular and molecular events that occur during pyroptosis.

**Figure 3:**
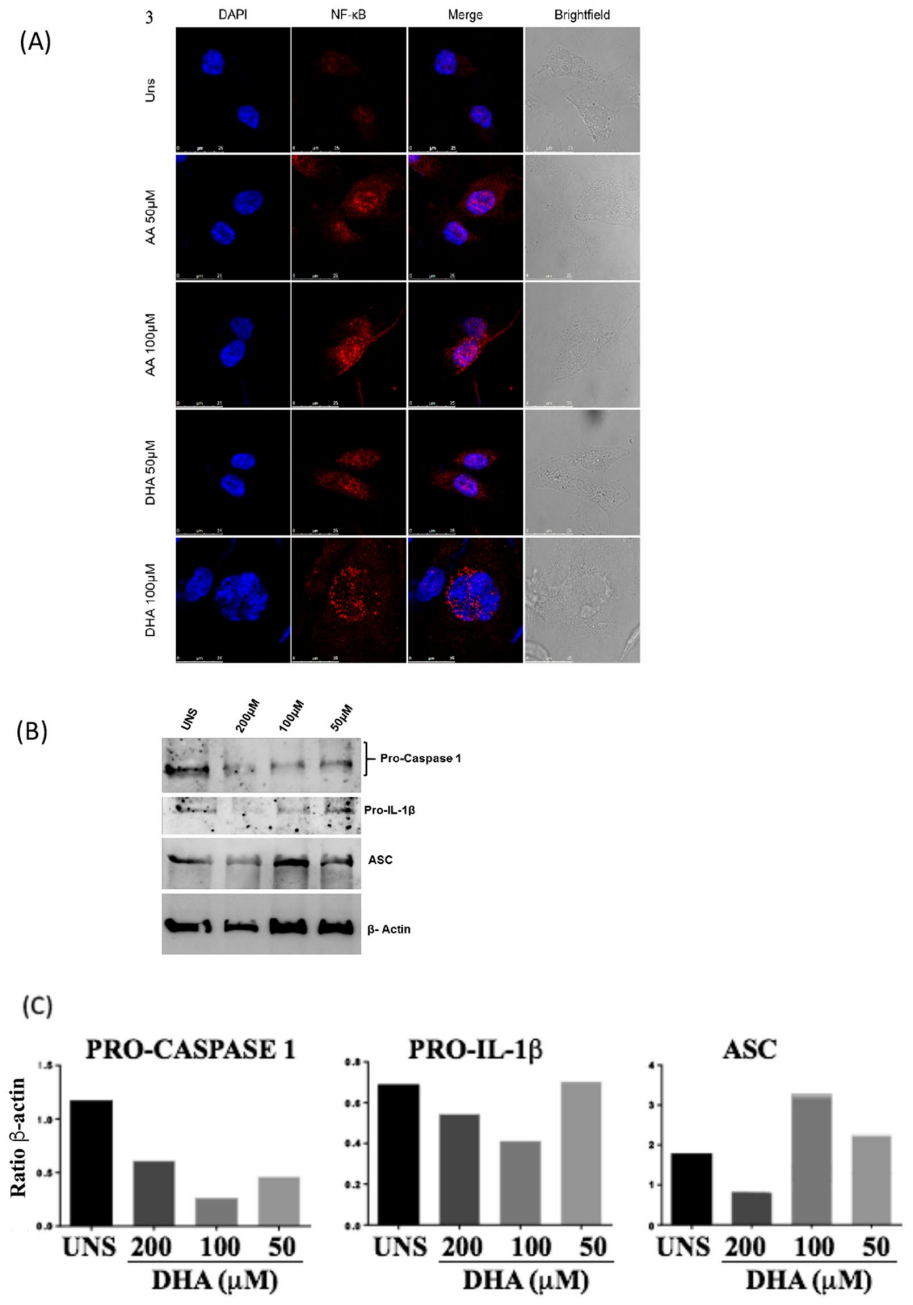
- DHA triggered priming of inflammasome signaling pathway. Breast cancer MDA-MB-231 cells were stimulated with 50 or 100μM of DHA or AA (**A**) for 18 h and NFκB translocation towards to nucleus was analyzed by confocal microscopy (NF-κB in red and nuclei in blue) compared to unstimulated cells (UNS). MDA-MB-231 cells were also stimulated with 50, 100 or 200μM of DHA (**B**) for 6 h and expression of pro-caspase-1, pro-IL-1β and adaptor protein ASC was analyzed by western blotting and bands densitometry was performed using Image J software (**C**).

**Figure 4:**
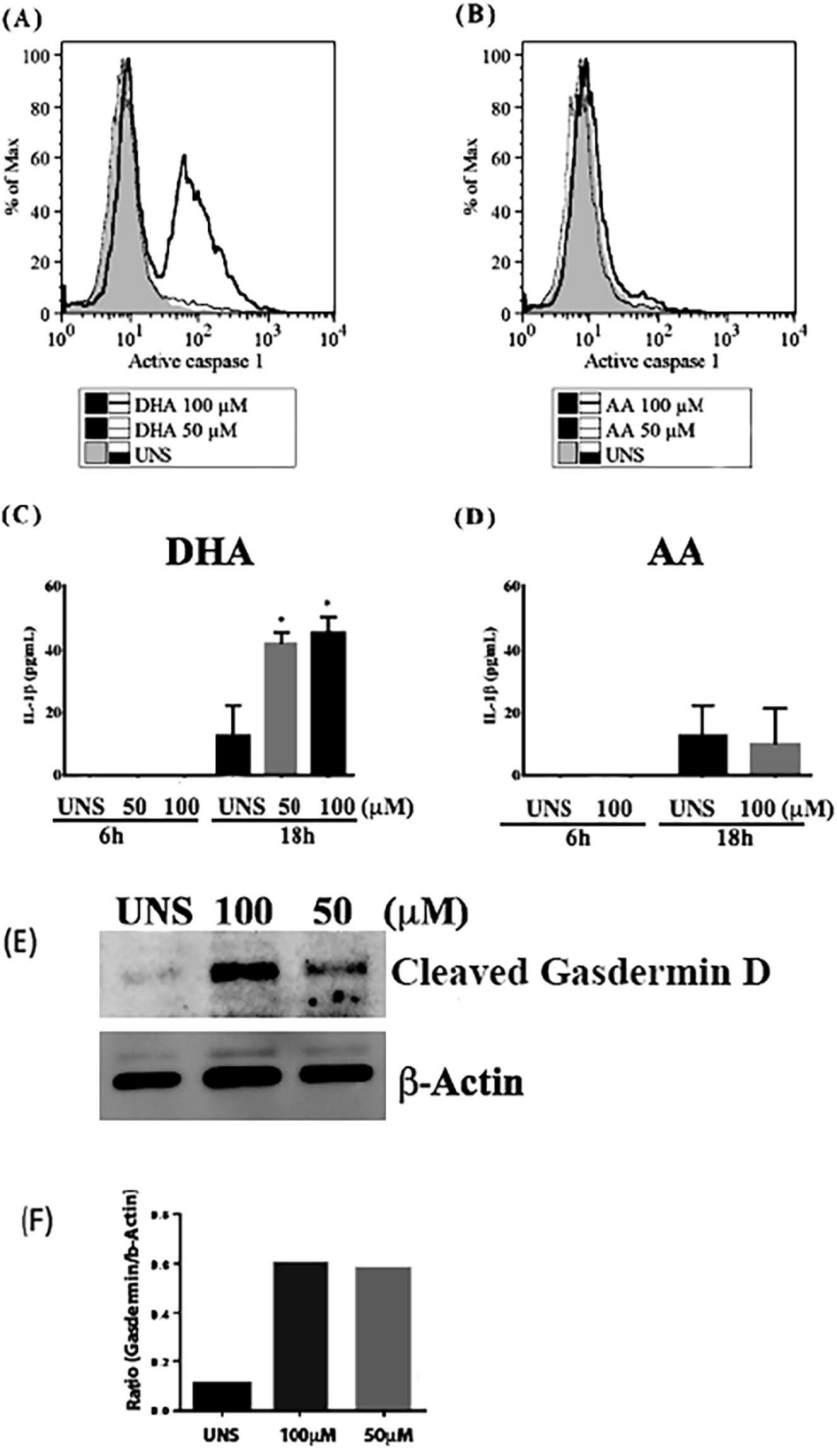
- DHA induced Caspase-1 and Gasdermin D activation and IL-1β secretion in MDA-MB-231. Cells were stimulated at the indicated concentrations of DHA (**A**) or AA (**B**) for 6 h and the amount of active caspase-1 was assessed by labeling cells with FAM-YVAD-FMK FLICA followed by flow cytometry analysis. Histograms are representative of three independent experiments. Cells were stimulated at the indicated concentrations of DHA (**C**) or AA (**D**) for 6 or 18 h and IL-1β secretion was assessed by ELISA. Each bar represents the mean concentration ± SD. *p < 0.05 compared to UNS (unstimulated cells). Cells were also stimulated at the indicated concentrations of DHA (**E**) for 3 h and active Gasdermin D was assessed by western blotting analysis. Bands densitometry was performed using Image J software (**F**).

We first analyzed the NF-κB translocation in breast cancer cells stimulated with DHA. After 18 h of treatment, DHA and AA100 μM induced NF-κB translocation from the cytoplasm to the nucleus of MDA-MB-231 (Fig. 3A). We also analyzed whether DHA can modulate the expression of pro-caspase-1, pro-IL-1β and inflammasome adapter protein ASC. DHA at 100 and 200 μM triggered an increase of ASC protein expression and a decrease of pro-caspase-1 and pro-1IL-1b after 6 h of stimulation (Fig. 3B,C), suggesting that both pro-caspase-1 and pro-IL-1β could have been cleaved and/or activated at this point. To address this question, we analyzed caspase-1 activation by staining cells with FAM-YVAD-FMK FLICA that binds specifically in activated caspase-1 forms. DHA but not AA at 100 μM increased the levels of active caspase-1 in MDA-MB-231 after 6 h of treatment (Fig. 4A and B). IL-1β levels were also analyzed in the cell supernatant and none of the fatty acids induced IL-1β secretion at 6 h of treatment. However, after 18 h of treatment, DHA significantly increased IL-β secretion (Fig. 4C,D).

Since caspase-1 mediates pyroptosis cell death by triggering gasdermin D activation, we analyzed if DHA could trigger gasdermin D cleavage in breast cancer cells MDA-MB-231. DHA at both 50 and 100 μM induced an increase of active gasdermin D after 3 h of stimulation (Fig. 4E,F).

**Figure 5:**
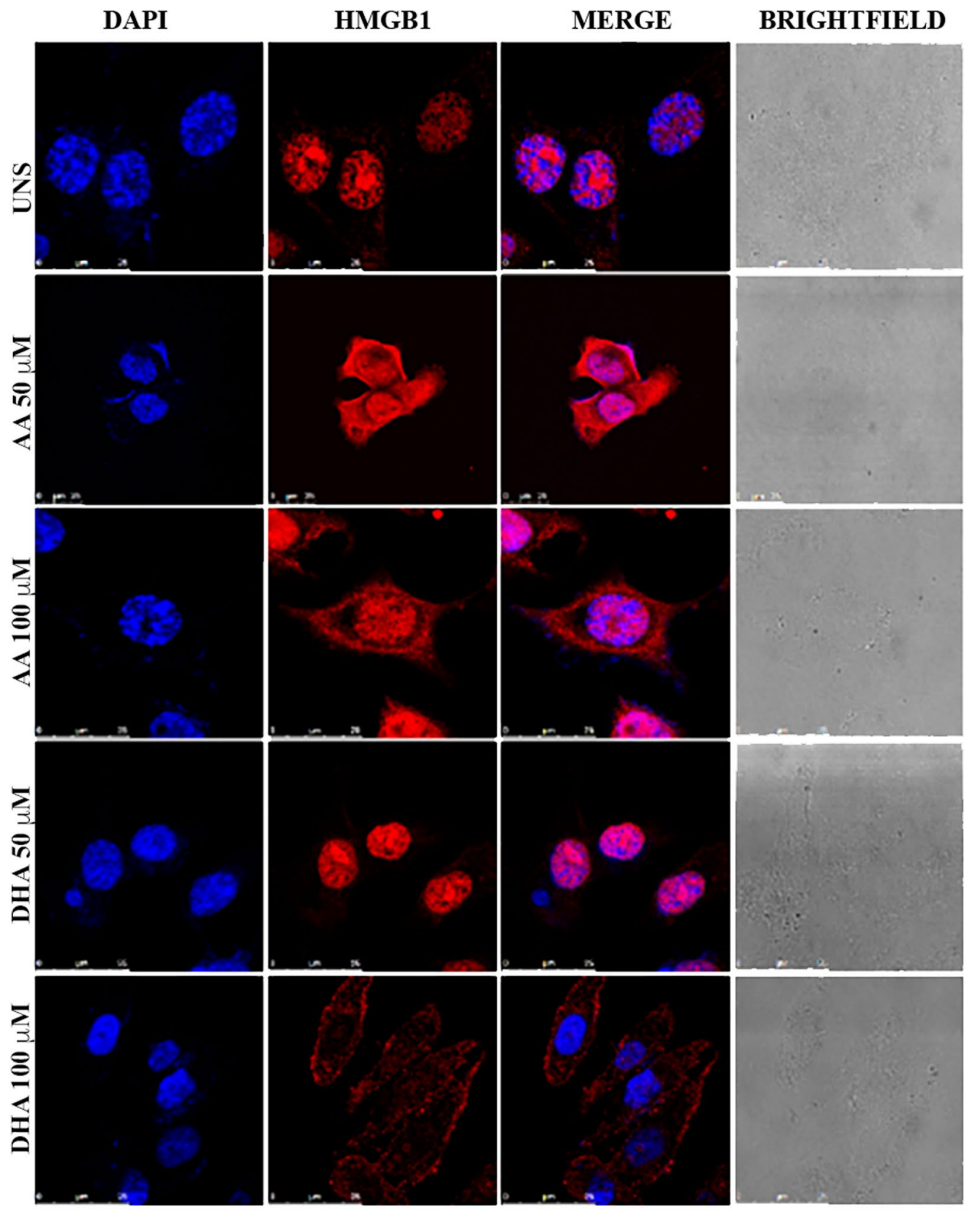
**–** DHA induced HMGB1 translocation towards the cytoplasm in breast cancer cells MDA-MB-231. MDA-MB-231 cells were stimulated with the indicated concentrations of DHA or AA for 18 hours. Immunofluorescence staining was performed to analyze HMGB1 cellular localization by confocal microscopy. This figure shows fluorescence microscopy analysis with HMGB1 in red and nuclei in blue. UNS: Unstimulated cells.

We also analyzed if DHA could trigger HMGB1 translocation in breast cancer cells. We observed that DHA induced HMGB1 translocation from the nucleus towards the cytoplasm of MDA-MB-231. AA had similar effect, but in a lesser extent since some HMGB1 remained in the nucleus (Fig. 5).

**Figure 6:**
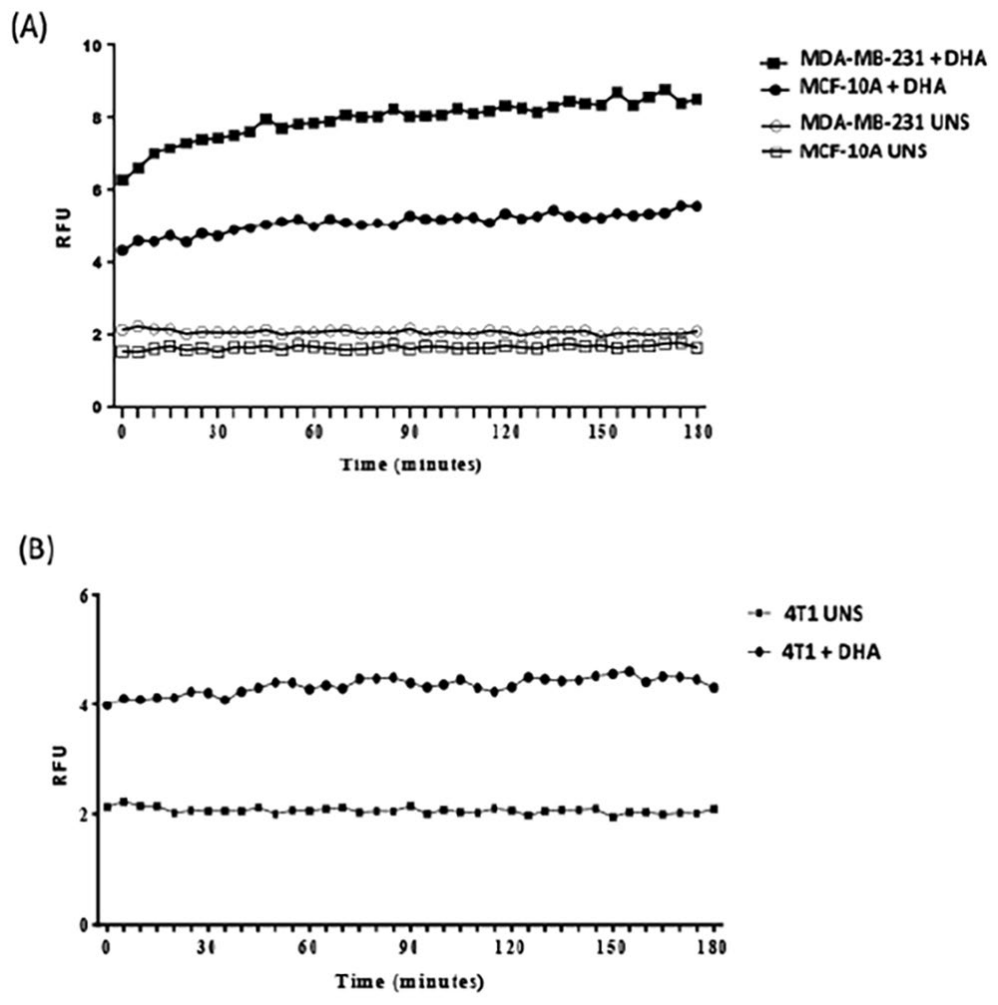
**-** DHA triggered membrane pore formation in breast cancer cells MDA-MB-231 and 4T1. MDA-MB-231 and MCF-10A (**A**) or 4T1 (**B**) cells were stimulated at the indicated concentrations of DHA (**A**) for 3 hours and stained with propidium iodide. Data are representative of three independent experiments (n = 5 each). UNS: unstimulated cells.

Considering that the most important event of pyroptosis is membrane pore formation, we analyzed whether DHA can cause the establishment of pores in breast cancer cell membranes. Our results showed that human breast cancer cells MDA-MB-231 stimulated with DHA presented a significant enhanced membrane pore formation after 3 h of stimulation when compared to unstimulated cells (Fig. 6A). This effect was much higher in breast cancer cells than non-cancerous cells MCF-10A (Fig. 6A). Similarly, murine breast cancer cells 4T1 treated with DHA showed an increase in membrane pore formation compared to unstimulated cell (Fig. 6B).

### DHA-induced pore membrane formation depended of inflammasome activation

**Figure 7:**
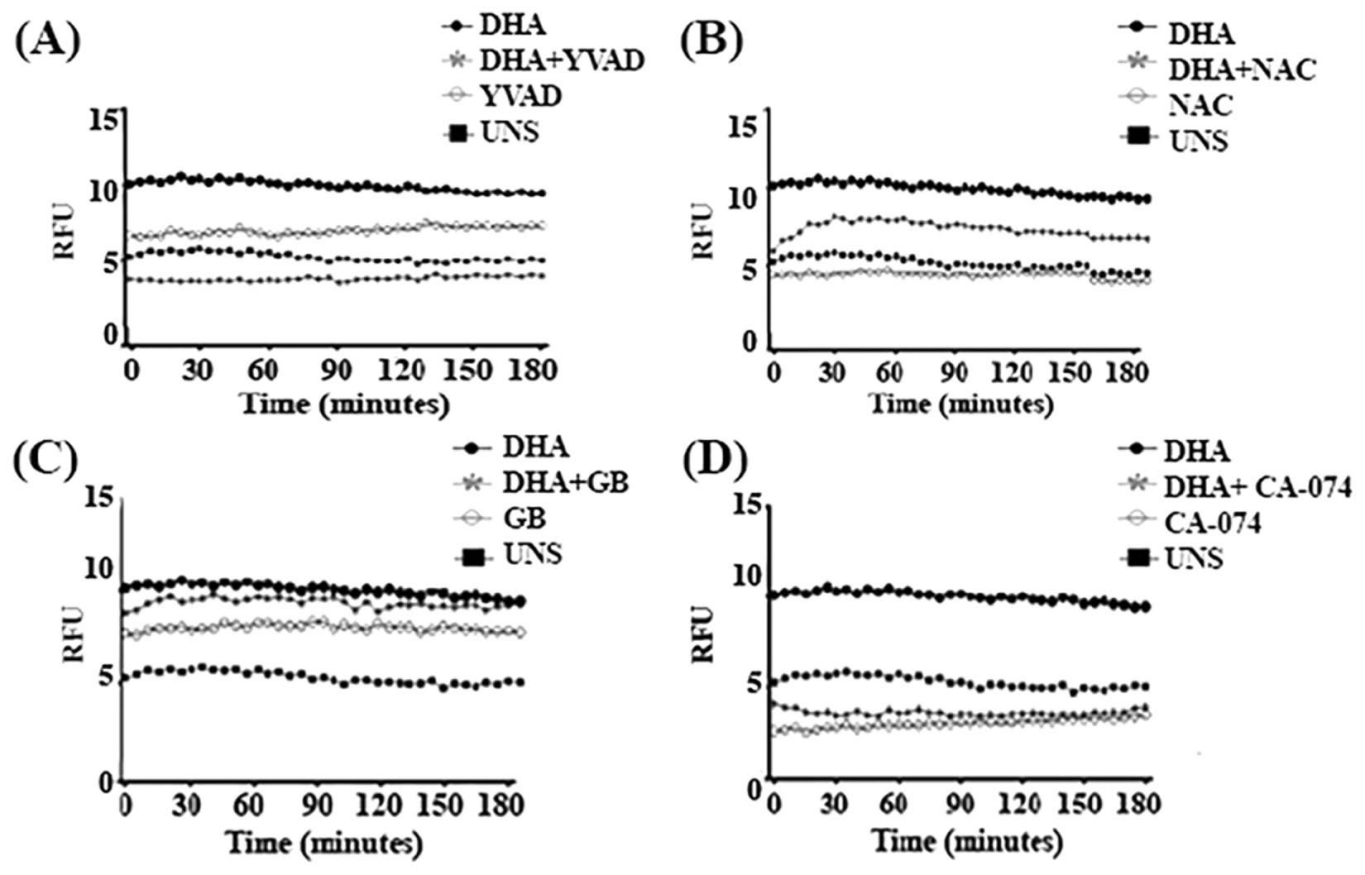
**–**DHA induced membrane pore formation dependent on caspase-1 activation, lysosomal damage and ROS formation. MDA-MB-231 cells were pre-treated for 1 h with caspase-1 inhibitor Ac-YVAD-cmk 100 μM (**A**) or ROS inhibitor N-acetyl-cysteine NAC 5 mM (**B**), potassium efflux inhibitor glyburide GB 150 µM (**C**) and CA-074, a lysosomal cathepsin B inhibitor 50 µM (**D**). Then, cells were stimulated with 100 µM DHA for 180 minutes and propidium iodide uptake was measured by spectrophotometry. Graphics are representative of three independent experiments (n = 5 each). UNS: Unstimulated cells. RFU: Relative fluorescence units.

To characterize the possible mechanisms involved in DHA-induced membrane pore formation in breast cancer cells MDA-MB-231, those cells were pre-treated with different inhibitors and pore formation was analyzed. Pre-treatment of cells with caspase-1 inhibitor YVAD at 100 μM decreased DHA-induced membrane pore formation (Fig. 7A). Likewise, pre-treatment of breast cancer cells with CA-074 and NAC significantly reduced membrane pore formation when compared to untreated cells stimulated with DHA alone (Fig. 7B,D), indicating that lysosomal damage and ROS formation may participate in this event. However, pre-treatment of breast cancer cells with glyburide did not affect DHA-induced membrane pore formation in MDA-MB-231 cells, suggesting potassium efflux are not necessary for this event (Fig. 7C).

## Discussion

In the present study, we hypothesized whether the cellular cytotoxicity induced by omega-3 DHA could be mediated by pyroptosis cell death in breast cancer cells. We have demonstrated here that DHA but not AA can induce pyroptosis cell death in triple-negative MDA-MB-231 breast cancer cells. Our results demonstrated that DHA led to NF-κB translocation, caspase-1 and gasdermin D activation, IL-1β secretion, HMGB1 translocation, pore membrane formation and loss of membrane integrity in MDA-MB-231 cells. All of these events are considered pyroptosis cell death-associated parameters and they are in accordance to the current working hypothesis suggesting that active caspase-1^42^, secreted HMGB1, gasdermin D cleavage, IL-1β release and membrane pore formation are indicators of pyroptosis induction^43^. Several investigators reported *in vitro* anticancer effects of this fatty acid on breast cancer^8–12^, however none work evaluated the occurrence of pyroptosis cell death in these cells.

Polyunsaturated fatty acids can be cytotoxic to different cancer cell types^8,44,45^. Our results in MDA-MB-231 and 4T1 breast cancer cells show that DHA reduced their viability within 24 hours whereas it had no significant effect on human non-cancerous mammary epithelial cells MCF-10A or PBMCs, suggesting that this fatty acid was cytotoxic only to cancer cells. Xue and colleagues^46^ showed that DHA strongly inhibited *in vitro* cell growth, and induced G1 cell cycle arrest both in 4T1 mouse breast cells and MCF-7 human breast cells, suggesting DHA has similar anti-cancer effect on both human and murine breast cancer cells. Other studies used different concentrations of DHA and also observed that it did not affect the viability range of human mammary epithelial cells MCF-10A at 24h^47^ and 96h^48^.

Both DHA and AA decreased MDA-MB-231 cell viability only at 200 μM. Therefore, for the mechanistic studies, we used the 100uM concentration for both fatty acids, since this concentration was cytotoxic for breast cancer cells but did not arrest cell viability. Corsetto and colleagues^49^ also showed that DHA and AA can reduce MDA-MB-231 cell viability but, in their study, AA only had an effect with 250 μM at 48 hours. Arachidonic acid is associated with tumor growth^16^ and tumor progression^17^, however its accumulated unesterified form in the cytoplasm can be cytotoxic and lead to cell death^50^.

The DHA-induced decrease in breast cancer cells viability occurred concomitantly with the increase in necrosis at 24 hours. Increased cell death percentage by necrosis started at 50uM and had a stronger action at 100 μM DHA, suggesting a clearly potentiated action when compared to unstimulated cells. These results corroborate with others studies that showed DHA-induced breast cancer cell cytotoxicity^9,11,49^.

Apoptosis is a widely described cell death pathway in the literature^51^, as well as its DHA-induced activation^9,11,49^. Apoptosis can occur concomitantly with other forms of cell death. It was described that one singular stimuli such as endoplasmatic reticulum stress^42^ in murine hepatocytes and like cytoplasmic DNA^52^ in murine macrophages could trigger both apoptosis and another type of cell death, the pyroptosis pathway.

Pyroptosis is an inflammatory and programmed cell death mediated by inflammasome and inflammatory caspases activation. Inflammasome protein complexes are canonically comprised of a sensor protein, the adaptor protein ASC (Apoptosis-associated Speck-like protein containing a CARD) and caspase-1. A non-canonical inflammasome pathway also involves caspase-11 (in mice) or caspase-4 and -5 (in humans). The requirement of inflammatory caspases in executing pyroptosis discriminates it from another necrotic and inflammatory form of programmed cell death which is accomplished independently of caspases called necroptosis^53–55^.

Inflammasome requires two signals for its activation. The first one involves a priming step that could be mediated by pattern recognition receptors (PRRs) which will lead to nuclear factors translocation towards the nucleus and transcription of important inflammasome pathway components such as pro-IL-1β, pro-caspase-1 and the common adaptor protein ASC. A second signal involves several cellular danger signals (DAMPs) such as reactive oxygen species generation, lysosomal damage, potassium efflux and ATP release. During bacterial infection or tissue damage, ATP is released from the intracellular compartments of both the host and bacterial cells into the extracellular milieu. The extracellular ATP, acting as a second signal for canonical NLRP3 inflammasome activation, induces pyroptosis in innate immune cells including macrophages. However, the signaling pathways regulating the pyroptosis are largely unknown, although some of which have been uncovered recently. The second inflammasome activation signals will lead to caspase-1 activation and maturation of pro-IL-1β into bioactive IL-1β^35,52^. Our data showed that DHA can activate inflammasome and induce IL-1β maturation and secretion in breast cancer cells. Contrarily, it has been demonstrated that DHA can inhibit inflammasome activation in immune cells such as macrophages. It suggests that DHA can differently regulate inflammasome activation depending on the type of cell^56,57^.

In our study, we demonstrated that DHA triggered high mobility group box 1 (HMGB1) translocation from the nucleus towards the cytoplasm after caspase-1 activation at 6 h. HMGB1 is an important DAMP, its secretion can occur after inflammasome activation^31^ and pyroptosis induction^58^. In our model, at 18 hours of DHA treatment, it was possible to see an almost complete HMGB1 translocation in MDA-MB-231 breast cancer cells. At the same concentration, AA-treated cells showed the presence of HMGB1 in the nucleus, differently of DHA-treated ones. Bell *et al*.^59^ were the first to indicate that this nuclear protein is also secreted by cells in late apoptosis. Thus, DHA-induced HMGB1 translocation may be due to the activated pyroptosis and can also be increased in late apoptosis.

DHA at 100 μM induced NF-κB nuclear translocation after 3 hours of treatment in MDA-MB-231 breast cancer cells. In addition, DHA at both 50 and 100 μM triggered an increase of ASC expression and a dose-dependent decrease of pro-IL-1β and pro-caspase-1 suggesting those important inflammasome proteins could be cleaved into its active forms at this point. This was later confirmed by our data that showed that DHA triggered caspase-1 activation and IL-1β secretion. This fatty acid increased the amount of active caspase-1 in MDA-MB-231 at 6 h, indicating the possibility of occurrence of inflammasome activation. We demonstrated here that breast cancer cells can present caspase-1 activation, and once activated, caspase-1 can process pro-IL-1β and induce its secretion. Other studies show that the interactions between caspases 1 and 5^60^ or caspases 1 and 4 also lead to increased pro-IL-1β activation^61^. Even though AA also induced NF-κB nuclear translocation, this fatty acid was not able to increase other pyroptosis-related parameters like active caspase-1 and secreted IL-1β, indicating that DHA-induced pyroptosis cell death triggered in breast cancer cells is a specific event.

It was recently identified a key substrate for inflammatory caspases called gasdermin D^62,63^, which upon its cleavage drives pyroptosis and is responsible for membrane pore formation, one of the most important events during pyroptosis. Therefore, we decided to investigate whether DHA could induce gasdermin D cleavage. We confirm this hypothesis by showing the expression of the gasdermin D N-terminal fragment (31 kDa) induced in MDA-MB-231 breast cancer cells treated with DHA. Gasdermin D can be cleaved by caspase-1 1^26,62,63^. More than a hundred proteins are thought to be cleaved by caspase-1, including caspase-7. Moreover, gasdermin D may contribute to IL-1b release by triggering membrane pore formation or regulating characterized IL-1β secretion mechanisms^64^.

Membrane rupture is the final event of pyroptosis cell death^32^. Here, we analyzed the membrane integrity by investigating the membrane pore formation in breast cancer cells treat with DHA. Our results showed that both human breast cancer cells MDA-MB-231 and murine mammary gland cancer cells 4T1 stimulated with DHA presented a significant enhanced membrane pore formation after 3 h of stimulation. This effect was cancer-specific since the same intensity effect was not observed in non-cancerous cells MCF-10A.

Our results suggest that DHA can induce pyroptosis by activating some inflammasome-dependent pathways. To confirm this effect, we analyzed pore membrane formation since it is an initial event of membrane rupture^32^, in the presence of different inflammasome pathway inhibitors. DHA-induced pore membrane formation was reduced in the presence of inflammasome activation inhibitors, indicating that this fatty acid can modulate pore membrane formation. Our results showed that caspase-1 inhibitor at 100 μM could protect cancer cells from pyroptotic cell death induced by DHA. Moreover, DHA could signal through ROS and lysossomal damage to induce pores in the cell membrane. However, potassium efflux appears to have no effect in this event.

Sagulenko *et al*. described that the NLRP3 inflammasome activates both pyroptosis and apoptosis, in murine macrophages, depending on the dose of the stimulus as well as on the time of treatment. This corroborates the importance of inflammasome responses in cell types expressing caspase-1, but further studies are needed to confirm the connecting between these two types of cell death^52^.

Finally, our study demonstrates a novel cell death pathway induced by DHA on breast cancer cells and pointed pyroptosis as a new and prominent target to mediate anti-cancer treatments. Thus, omega-3 DHA fatty acid can trigger pyroptosis in MDA-MB-231 triple-negative breast cancer cells and this finding sheds new light on the anticancer effect of DHA, which may have an important roleomega-3 supplementation in cancer therapy.

## Methods

### Cell culture and stimulus

In this study, we used triple-negative human breast cancer cells MDA-MB-231, 4T1 murine breast cancer cells, human normal epithelial mammary gland cellsMCF-10A, and human peripheral blood mononuclear cells PBMCs. Those cells were cultivated and analyzed after DHA or AA fatty acid stimulation. The University of Brasilia Ethics Committee approved this research and all participants provided written informed consent.

MDA-MB-231 and MCF-10A cells were kindly provided by José Raimundo Corrêa (University of Brasilia, Brasília, BRA). MDA-MB-231 cells were cultivated in L-15 medium (Sigma Chemical Company, St. Louis, USA) supplemented with 10% fetal bovine serum (FBS, GIBCO, Gaithersburg, USA) and 1% antibiotic-antimycotic (GIBCO) at 37 °C. MCF10A cells were cultured in DMEM/Ham’s F-12 (GIBCO-Invitrogen, Carlsbad, CA) supplemented with 100 ng/ml cholera toxin, 20 ng/ml epidermal growth factor (EGF), 0.01 mg/ml insulin, 500 ng/ml hydrocortisone, and 5% chelex-treated horse serum. The 4T1 mouse mammary tumor cell line was cultured in high glucose DMEM supplemented 5% FBS, and antibiotics (100 units/ml penicillin and 100 μg/ml streptomycin) at 37 °C in a humidified atmosphere containing 5% CO2. All the growth factors were purchased from Sigma (St. Louis, MO, USA).

Blood samples from healthy individuals were collected after their informed consent. Peripheral blood mononuclear cells (PBMCs) were isolated by density gradient centrifugation using Histopaque 1077 (Sigma). Isolated PBMCs were cultured in RPMI 1640 medium (GIBCO), supplemented with 10% FBS and 1% antibiotic-antimycotic at 37 °C with 5% CO_2_.

Docosahexaenoic acid (DHA) and arachidonic acid (AA) were purchased from Sigma and diluted in dimethyl sulfoxide (DMSO, Sigma) and absolute ethanol (J.T.Baker®) respectively. Cells were stimulated with them as described in the text. Lipopolysaccharide (LPS, Sigma) and adenosine triphosphate (ATP, Sigma) were used as controls of inflammasome induction. To analyze membrane pore formation signaling pathway, we used the following compounds: Glybenclamide (GB, Sigma), KCl (Sigma), N-Acetyl cysteine (NAC, Sigma), CA-074 (Sigma) and Z-VAD and Ac-YVAD-CHO (EnzoLife Sciences).

### MTT assay

Cells were plated in triplicate wells in 96-well plates and stimulated or not with the fatty acids. 3-(4,5-Dimethyl-thiazol-2-yl)-2,5-diphenyl-tetrazolium bromide (MTT, Sigma) was added to each well at 10% (5 mg/ml MTT in phosphate buffered saline). The plates were incubated at 37 °C for 3 hours and then processed as described previously^65^. We also measured the concentration of DHA or AA which gives 50% of the maximum cytotoxicity effect, the (EC50).

### Flow cytometric analysis

Apoptosis and necrosis were measured using Dead Cell Apoptosis Kit with annexin V FITC and PI (Thermo Fisher, Waltham, USA) and active caspase-1 was detected with FAM-FLICA™ Caspase-1 Assay Kit (Immunochemistry, Bloomington, USA), both according to manufacturer’s protocol. Cells were analyzed on a FACS Calibur flow cytometer (BD Biosciences, East Rutherford, USA), and flow data was analyzed with the FlowJo v.7.6.5 (Tree Star, Inc., Ashland, USA).

Cell membrane integrity was measured by flow cytometry with propidium iodide (PI, Enzo Life Sciences, New York, USA) staining. MDA-MB-231 cells were plated and stimulated or not with the fatty acids. Supernatants were centrifuged at 3000 G for 5 minutes to pellet the cells. Adhered cells in the plate were dissociated with trypsin (GIBCO) and transferred to the tubes with pellets. Each tube was stained with 0,5 μL of PI (100 µg/ml) and incubated for 10 minutes in the dark before analysis on the flow cytometer.

### Immunofluorescence staining

Cells were grown on glass coverslips and stimulated or not with the fatty acids. They were fixed with 4% paraformaldehyde and permeabilized with 0.2% Triton X-100 buffer. The cells were then incubated with anti-HMGB1 (Sigma) or anti-NF-κB (Santa Cruz Biotechnology, Dallas, USA) rabbit monoclonal antibody (1:200) overnight at 4 °C. Bound antibody was detected with Alexa 488-labeled anti-rabbit IgGF(ab’)2 (Thermo Fisher) (1:2000). Nuclei were stained with DAPI (Sigma) (1:5000) for 5 minutes at room temperature. Samples were visualized using the fluorescence microscope Leica TCS SP5 (Leica Microsystems, Wetzlar, DEU).

### ELISA

Supernatant IL-1β concentration was detected by ELISA with a R&D Systems (USA) kit. Microtiter plates were coated overnight at room temperature with capture antibody and blocked with Reagent Diluent for 1 hour. Serially diluted samples were added to the wells in triplicate and incubated overnight at 4 °C. After extensive washing, the cells were incubated with detection antibody and then Streptavidin-HRP. After washing, substrate solution was added and the plates were incubated for 15 minutes at room temperature. Plates were read after adding stop solution at 450 nm using SpectraMax M3spectrophotometer (Molecular Devices, Sunnyvale, USA).

### Pore formation assay

Pore formation was determined by quantification of propidium iodide (PI) uptake. Cells were plated in triplicate wells in black 96-well plates. Before stimulation, the media were replenished with RPMI 10% without phenol red, 0. 38 g/L NaHCO3, 20 mM HEPES and 3 µg/ml PI. The plates were incubated at 37 °C in a SpectraMax M3 spectrophotometer (Molecular Devices, Sunnyvale, USA) and propidium iodide was excited at 538 nm.The fluorescence emission was read at 617 nm at every 5 minutes.

### Western blotting analysis

Proteins were extracted using Lysis Buffer (Tris-HCl 50 mM, NaCl 150 mM, EDTA 5 mM and Triton-X100 1%) and Cocktail Protease Inhibidor (04693159001, Roche). 14% polyacrilamide gelswere transferred to PVDF membrane using a semi-dry system. The membrane was blocked for 1 hour and incubated overnight at 4 °C with the primary antibody (Anti-pro-caspase-1, Gasdermin D, pro-IL-1β and ASC; Abcam). The membrane was then incubated for 1 hour with secondary antibody (Jacksons Immuno Research). The anti-β-actin antibody (Aldrich) was used as loading control. The bands were revealed using Chemiluminescent Substrate (Westar Supernova XLS3L and XLS3P) by Image Quant LAS 4000 (GE Healthcare Life Sciences). The bands were analyzed with the ImageJ software (Version 1.8) for densitometry analysis.

### Statistical analysis

Statistical analysis was performed with GraphPad Prism 6.0 (GraphPad Software Inc. San Diego, USA). One-way or two-way ANOVA tests were used followed by Tukey’s post-test. *P* < 0.05 was considered statistically significant.

All methods were performed in accordance with the relevant guidelines and regulations of University of Brasilia (Brazil) were this work took place.
