# Author Correction: Omega-3 docosahexaenoic acid induces pyroptosis cell death in triple-negative breast cancer cells

**DOI:** 10.1038/s41598-018-27850-y

**Published:** 2018-06-22

**Authors:** Nathalia Pizato, Beatriz Christina Luzete, Larissa Fernanda Melo Vasconcelos Kiffer, Luís Henrique Corrêa, Igor de Oliveira Santos, José Antônio Fagundes Assumpção, Marina Kiyomi Ito, Kelly Grace Magalhães

**Affiliations:** 1 Department of Nutrition, University of Brasilia, Brasilia, 70910–900 Brazil; 2 Laboratory of Immunology and Inflammation, University of Brasilia, Brasilia, 70910-900 Brazil

Correction to: *Scientific Reports* 10.1038/s41598-018-20422-0, published online 31 January 2018

The Acknowledgements section in this Article was omitted. The Acknowledgements section should read:

“This research is supported by funding agency FAPDF.”

